# OA06 Catastrophic anti-phospholipid syndrome presenting as stroke in a young male: diagnostic and therapeutic challenges in a resource-limited setting

**DOI:** 10.1093/rap/rkag113.006

**Published:** 2026-11-03

**Authors:** Hadi Rabee, Alaa Shamlawi

**Affiliations:** Al-Watani Governmental Hospital, Nablus, Palestine; Surrey and Sussex Healthcare NHS Trust, Surrey, United Kingdom; Al-Watani Governmental Hospital, Nablus, Palestine

## Abstract

**Introduction:**

Catastrophic antiphospholipid syndrome (CAPS) is a rare life-threatening variant of antiphospholipid syndrome (APS), characterised by rapidly progressive thrombosis involving multiple organs. Early recognition is crucial, as delayed diagnosis is associated with significant morbidity and mortality. CAPS may occur as a primary condition or secondary to systemic lupus erythematosus (SLE), and can occasionally represent the first manifestation of SLE. Diagnosis is particularly challenging in resource-limited settings, where access to rheumatologists, specialised investigations and advanced therapies may be restricted. This case highlights the importance of maintaining a high index of suspicion for CAPS in young patients presenting with thrombosis and multi-system involvement.

**Case description:**

A 30-year-old man with unremarkable past medical history presented with sudden-onset left-sided upper and lower limb weakness, associated with frontal headache and slurred speech. CT and MRI scans showed a large acute non-haemorrhagic infarction in the right fronto-parietal lobes, along with multiple lacunar infarctions. Further evaluation revealed evidence of multisystem involvement, including diffuse ST-segment elevation on electrocardiography with elevated cardiac biomarkers but non-obstructed coronaries on angiography, elevated inflammatory markers, prolonged activated partial thromboplastin time, haemolytic anaemia with positive Coombs test, and acute kidney injury with active urine sediment (Table 1).

Multisystem involvement occurring simultaneously prompted consideration of a systemic thrombotic process. Autoimmune investigations demonstrated persistent triple positive antiphospholipid antibodies, and positive ANA and anti-dsDNA antibodies. A kidney biopsy was not available at our centre. The constellation of acute cerebral, cardiac and renal manifestations fulfilled criteria for probable CAPS secondary to SLE.

Management required collaboration between internists and nephrologists; no rheumatologist was available. Treatment consisted of high-dose intravenous methylprednisolone, rituximab and hydroxychloroquine. The patient demonstrated significant improvement in neurological function, and normalisation of the kidney function and cardiac biomarkers. This case illustrates the diagnostic complexity of CAPS and underscores the importance of early recognition and treatment, even in a resource-limited setting.

**Discussion:**

This case demonstrates several important challenges in the diagnosis and management of APS and CAPS. Stroke in a young adult often prompts investigation for cardioembolic, vascular or inherited thrombophilia causes; however, the coexistence of cardiac injury, renal dysfunction, haemolytic anaemia and coagulation abnormalities suggested a broader systemic process. Recognition of this pattern was critical in identifying probable CAPS before irreversible organ damage occurred.

The case is notable because CAPS represented the initial manifestation of previously undiagnosed SLE in a young male patient. Both SLE and APS are more frequently reported in women, which may reduce clinical suspicion in male patients and contribute to diagnostic delay. The simultaneous involvement of multiple organs within a short timeframe further supported the diagnosis of CAPS.

Management decisions were influenced by the realities of a resource-limited environment. In many settings, availability of rheumatologists or haematologists, access to plasma exchange, intravenous immunoglobulin, complement inhibition and advanced organ specialist testing may be limited. Consequently, treatment strategies often rely on prompt clinical recognition (based on a probable rather than a definite APS diagnosis) and the judicious use of available immunosuppressive and supportive therapies. This case highlights the need for flexible, pragmatic decisions and management pathways that can be implemented outside highly specialised centres, as these were not available in the country, during a specific time period (COVID-19 pandemic era).

The case also raises important questions regarding optimal treatment escalation where access to guideline-recommended therapies is restricted. It provides an opportunity to discuss how clinicians can balance diagnostic certainty against the need for urgent intervention in rapidly progressive life-threatening autoimmune thrombotic disease. Ultimately, the patient’s favourable neurological recovery reinforces the importance of early multidisciplinary management and consideration of APS/CAPS in young patients presenting with stroke and evidence of multisystem disease.

**Key learning points:**

1. Catastrophic antiphospholipid syndrome should be considered in young patients presenting with stroke accompanied by evidence of multisystem organ involvement.

2. APS and CAPS may represent the first manifestation of systemic lupus erythematosus and can occur in male patients despite the strong female predominance of these conditions.

3. Simultaneous neurological, cardiac and renal involvement should prompt urgent evaluation for systemic thrombotic and autoimmune disorders.

4. Prolonged activated partial thromboplastin time does not exclude a prothrombotic condition and should raise suspicion for antiphospholipid antibodies in the appropriate clinical context.

5. Early recognition and treatment are essential to prevent irreversible organ damage and improve outcomes in CAPS.

6. Resource limitations may restrict access to advanced diagnostics and therapies; however, timely clinical assessment and multidisciplinary decision-making remain critical components of care.

7. Clinicians working in low-resource settings require practical approaches to diagnosing and managing APS/CAPS when guideline-recommended investigations/interventions are not readily available.

**Learning objectives for conference discussion include:**

1. Optimising diagnostic pathways for suspected CAPS in resource-constrained environments.

2. Identifying triggers for early rheumatology involvement in young patients with thrombotic events.

3. Understanding treatment prioritisation when plasma exchange, IVIG or other advanced therapies are unavailable.

4. Sharing experience regarding long-term management and secondary thrombosis prevention in APS associated with SLE presenting with large ischaemic strokes.

**OA06 Table 1:** Significant test results for the patient at presentation.

Test	Result/Unit	Normal Range
Haemoglobin	72 g/L	125–185
Activated partial thromboplastin time	46 seconds	25-35
Erythrocyte sedimentation rate	125 mm/hour	0-10
C-reactive protein	3 mg/dl	0-0.5
Creatinine	145 mmol/L	64–104
Anti-nuclear antibody (ANA)	1:80	<1:40
Anti-double stranded DNA (dsDNA) titre	98 IU/ml	0-10
Complement C3	85 mg/dl	90-180
Complement C4	15 mg/dl	20-50
Urinalysis	+3 protein, +2 blood	No blood or proteins
Urine protein to creatinine ratio	55 mg/mmol	<15 mg/mmol

